# Tritium contamination and hydrological transport in the Shagan River: An isotope hydrology study

**DOI:** 10.1371/journal.pone.0333260

**Published:** 2025-10-09

**Authors:** Togzhan Toktaganov, Ainur Mamyrbayeva, Аssan Aidarkhanov, Almira Aidarkhanova, Almira Raimkanova

**Affiliations:** Institute of Radiation Safety and Ecology, National Nuclear Center of the Republic of Kazakhstan, Kurchatov, Kazakhstan; Universiti Teknologi Malaysia, MALAYSIA

## Abstract

This paper presents the results of a study of the distribution of the radionuclide tritium (^3^H) and stable isotopes of hydrogen and oxygen in the Shagan River. The Shagan River flows through the territory of the ‘Balapan’ test site and is the longest watercourse on the territory of the former Semipalatinsk Test Site (STS), and is also a left-bank tributary of the Irtysh River. The isotope hydrology method (^2^H and ^18^O) was used to determine the conditions for the formation of tritium-contaminated waters in the Shagan River. The study revealed certain sections of the river, especially at distances of 5, 10 and 14 km from the source, where elevated concentrations of tritium and stable isotopes indicated a significant influx of groundwater. A combined analysis of the isotopic composition and mineralization helped to assess the role of evaporation and identify hyporheic zones that contribute to the redistribution of radionuclides. At the confluence of the Shagan and Irtysh rivers, tritium activity does not exceed the intervention level of 7600 Bq/l recommended by the World Health Organization and the International Atomic Energy Agency for drinking water.

## 1. Introduction

Isotope hydrology is a section of hydrology that uses an isotopic composition of water to study the movement, distribution and storage of water in the environment. The term “isotopic composition of water” usually refers to the content of deuterium (^2^H) and oxygen-18 (^18^O), which are stable isotopes. Stable ^18^O and ^2^H are powerful tools for tracing and revealing sources and pathways of water resources contamination and the migration of aquatic medium contaminants [1,2].

In phase transitions (evaporation, freezing etc.), because different stable isotopes differ in mass, they may behave differently. During evaporation, lighter isotopes such as ^1^H and ^16^O evaporate more easily than the heavier ones such as ^2^H and ^18^O. This leads to a relative enrichment of the residual water by heavy isotopes to be measured as an increase in the heavy-to-light isotopic ratio. During condensation, heavy isotopes are typically retained in the vapor phase, which leads to a relative decrease in the content of heavy isotopes in the residual water. As a result of freezing, lighter isotopes are usually incorporated in ice thereby resulting in a relative enrichment of the residual water by heavy isotopes [3].

To assess the degree of water fractionation, a deuterium excess is used. A deuterium excess (d_excess_) is the amount of difference between concentrations of deuterium and oxygen-18 in the water. This index is a useful tool in the isotope hydrology upon which one can obtain a valuable information on the origin of waters, and which, when interpreted and combined with other parameters like salinity, allow for a fuller understanding of water-related processes [4].

The isotope hydrology technique or determination of stable water isotopes (^2^Н and ^18^О) has proved itself in operations mostly aiming to determine hydrological processes to monitor contamination of ground and surface waters [5,6]. From the hydrogeological perspective, stable isotopes were used in the work with a surface and groundwater exchange [7].

The migration of radionuclides in the aquatic medium is one of the most complicated processes [8,9]. Contamination of surface and groundwaters by radionuclides is related to nuclear tests, various accidents at nuclear fuel cycle facilities (for example, the Chernobyl NPP and Fukushima-1 NPP accidents, Kyshtym accident and others) and to leakages from underground radioactive waste (RAW) repositories [10–12]. Radionuclides, while getting into the water, behave differently depending on physical and chemical factors. Tritium is a dynamic radionuclide because it has a high migration capacity. This capacity points to a high transport of this radionuclide by surface and groundwaters [13–18].

STS was one of the largest sites to conduct nuclear tests, and its area is about 18,500 km². The more than 40 years of the Semipalatinsk Test Site activities are one of the stages in the armament race history of the former USSR. Altogether, over the STS operating period, 340 underground (‘Degelen’, ‘Balapan’ and ‘Sary- Uzen’ sites), 30 aboveground and 86 air (‘Experimental Field’ site) tests were conducted [19]. Due to nuclear testing in the territory of the former STS, the aquatic medium has also been exposed to radioactive contamination. The main source of radionuclides discharged outside the ‘Balapan’ test site is the Shagan River. Research into the migration of radionuclides via surface and groundwaters is of particular importance, being one of the priorities after the STS shutdown [20–23,24].

This paper aimed to identify migration features of ^3^Н across the length of the Shagan River. To study conditions under which contamination of waters by tritium is formed, operations were carried out to reveal any water exchange processes by means of an isotope hydrology technique.

## 2. Materials and techniques

The ‘Balapan’ site is located in the southeastern part of the test site occupying a 780 km^2^ area. This test location was designed for underground nuclear blasts in boreholes and for simulation experiments using conventional explosives. Altogether, 105 high yield nuclear tests of up to 150 kt were conducted here including the first excavation blast in the former USSR with soil ejected as part of the experiment to create an artificial water reservoir [25].

The Shagan river is the longest surface water stream flowing along the eastern boundary of the ‘Balapan’ site outside STS and is a left-bank tributary of the Irtysh river. In the course of earlier studies of this river, it was found that ^3^H is the main radionuclide polluting the waters of the Shagan. Numerical values of the concentration of ^3^Н activity in river water are constantly recorded and amount to about 300,000–350,000 Bq/kg in the 5 kilometer section downstream of the Atomic Lake and 100–200 Bq/kg where it flows into the Irtysh River [26,27].

### 2.1. Field work

A peak of radionuclide contamination of water bodies at the ‘Balapan’ site falls during August [28], therefore field observations and sampling at the ‘Balapan’ site were accomplished in August. In that period, the influence of snowmelt and flood waters on the concentration of man-made ^3^Н is eliminated.

**Fig 1:**
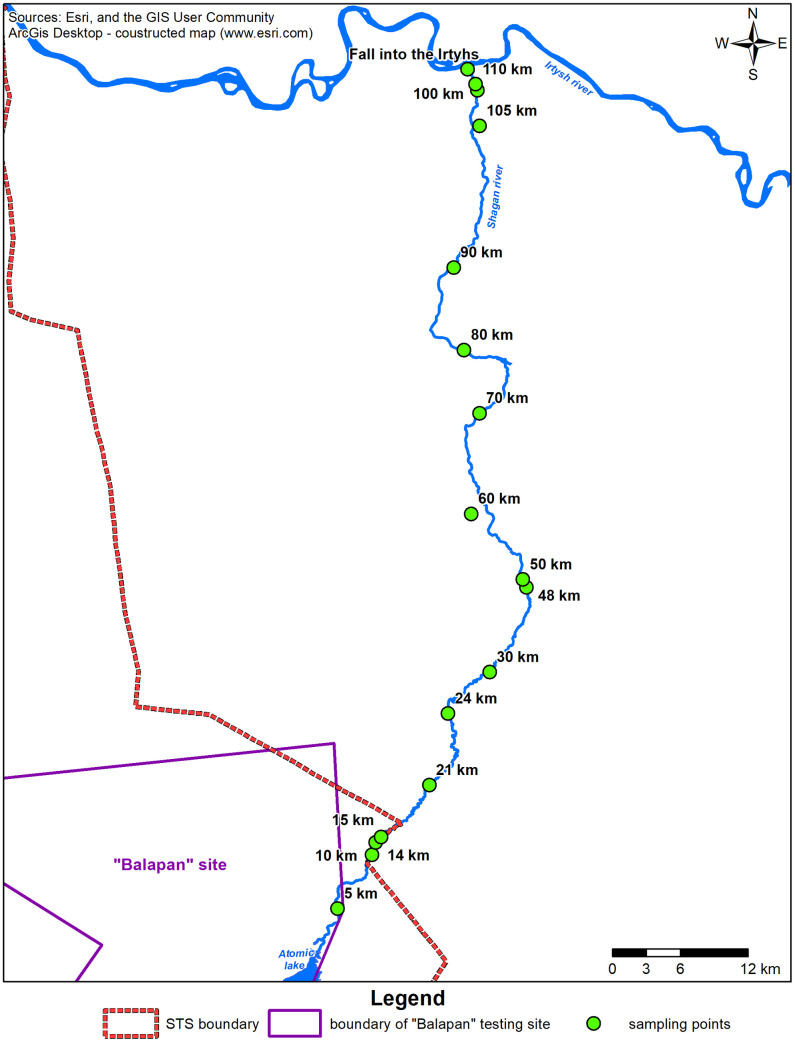
Water sampling points on the Shagan river and before it flows into the Irtysh river.

To understand the distribution of ^3^Н in the riverbed, water was sampled across the length from the ‘Atomic Lake’ as far as the inflow into the Irtysh River (Fig 1). The interval between sampling points was 5–10 km. The exception were sampling points at 14, 48 and 105 km due to seasonal drying up. Altogether, 17 samples from the riverbed were collected.

**Fig 2:**
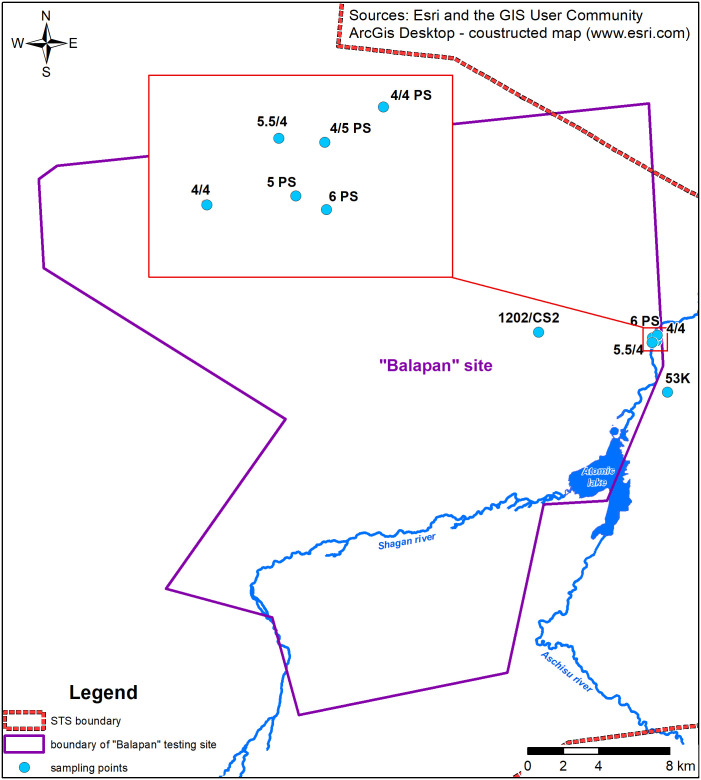
Sampling points of groundwaters.

Groundwaters were samples from monitoring wells in the vicinity of the Shagan River 5 km away from the ‘Atomic Lake’ where elevated values of ^3^Н are noted in the river water. Altogether, 8 samples were collected (Fig 2).

### 2.2. Laboratory analysis

Water samples were stored under refrigerated conditions until analysis. The isotopic composition (δ²H and δ¹⁸O) was determined using a high-sensitivity laser spectrometer LGR 912−0008 (Los Gatos Research, Inc.) [29]. Results were expressed in δ notation relative to international standards according to the equation:

$\delta =(\frac{{\text{R}}_{\text{sample}}}{{\text{R}}_{\text{standart}}}-\text{1})\times \text{1000}‰$,

where R_sample_ and R_standart_ represent the ²H/¹H and ¹⁸O/¹⁶O ratios in the sample and the reference, respectively. Standard Mean Ocean Water (SMOW, IAEA, Vienna) was used as the primary reference, while internal standards were calibrated against VSMOW [30]. Each water sample and reference standard were measured six times using a microliter syringe (S1 Fig). The final result was calculated as the mean of the three to four closest values, while outliers were excluded following. The analytical precision was ±1.0 ‰ for δ²H and ±0.5 ‰ for δ¹⁸O, consistent with previously reported values for the LGR 912-0008 [31,32].

The content of ^3^Н was determined by liquid scintillation spectrometry using а β-spectrometer TRI-CARB 2900 TR. Measurements were performed as per the standard procedure ISO 9698/2019 [33]. The measurement error was 10% at most.

## 3. Results and discussion

### 3.1. The results

#### 3.1.1. *Results of the isotopic analysis (*^*2*^*Н and* ^*18*^*О).*

Results of the isotopic analysis of the Shagan river are listed in Table 1.

**Table 1:** Results of the isotopic analysis of the Shagan River water.

Sampling points	Month of sampling	δ^2^H, ‰	δ^18^O, ‰	dexcess
**5 km**	september	−113,2	−14,5	−10,3
**10 km**	august	−90,4	−11,2	−10,9
**14 km**	september	−112,9	−14,4	−10,7
**15 km**	august	−81,8	−8,3	−22,9
**21 km**	august	−75	−7,5	−21,8
**24 km**	august	−70,8	−7,2	−19,7
**30 km**	august	−70	−6,5	−23,9
**48 km**	august	−69,8	−7	−20,3
**50 km**	august	−67,7	−7,3	−15,9
**60 km**	august	−66,7	−8,5	−6,4
**70 km**	august	−64,8	−7,9	−8,7
**80 km**	august	−80,4	−8,2	−22,2
**90 km**	august	−89,1	−11	−11
**100 km**	august	−89,7	−11,2	−10,2
**105 km**	august	−91,2	−11,2	−11,7
**110 km**	august	−98,2	−12,1	−12,3
**Inflow into Irtysh**	august	−92,3	−11,1	−13,5

According to findings of the in vitro analysis, δ^18^O in surface 168 waters of the Shagan River varies from −7.2‰ to −14.5‰, and δ^2^Н varies from −64.8‰ to −113.2‰.

In groundwaters, stable isotope δ^18^O varies from −14.6‰ to 16.6‰, the content of δ^2^Н varies from −113.1‰ to −120.9‰; the results are listed in Table 2.

**Table 2:** Results of the isotopic analysis of groundwaters.

Well number	Month of sampling	δ^2^H, ‰	δ^18^O, ‰	dexcess
53К	august	−120,9	−16,6	−3,2
5.5/4	august	−118	−16	−4,7
4/4	august	−113,7	−14,9	−7,6
1202-CS2	august	−113,1	−14,6	−9,7
5 PS	august	−114,9	−15,6	−3,9
4/5 PS	august	−115,8	−15,4	−6,7
4/4 PS	august	−114,3	−16	−0,7
6 PS	august	−115,6	−15,4	−6,3

#### 3.1.2. *Determinations of the content of man-made* ^*3*^*Н.*

**Fig 3:**
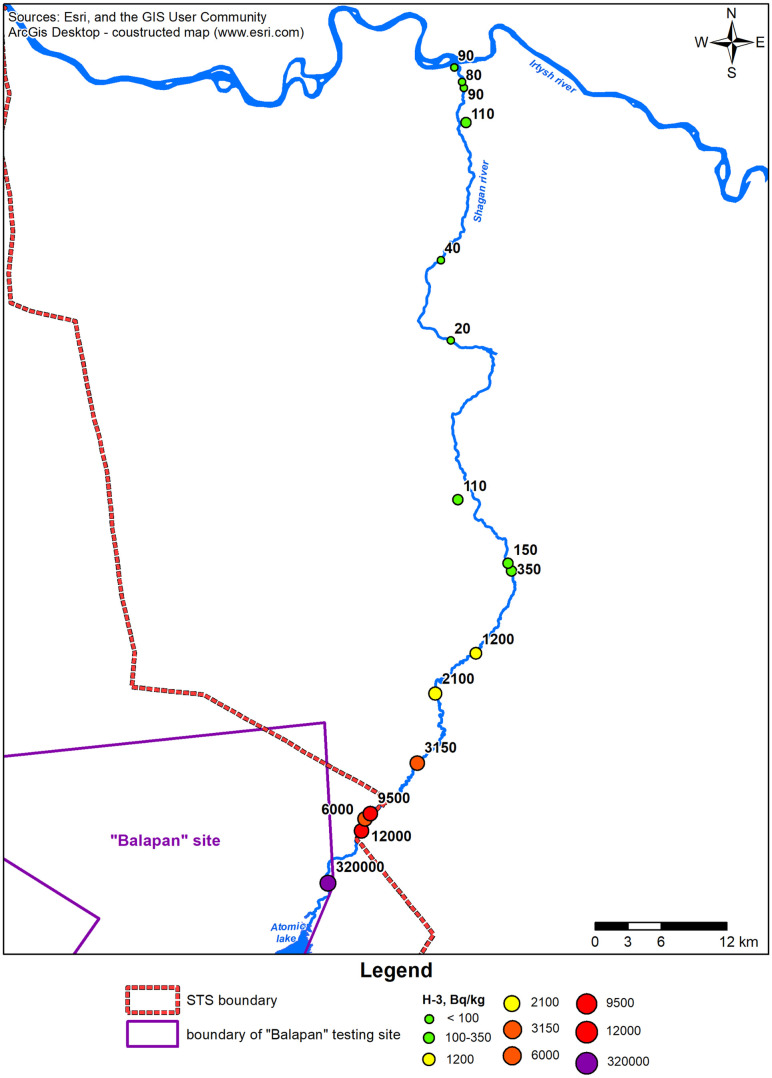
Beta-spectrometric measurements of the content of ^3^Н in the Shagan River water.

Determinations obtained for ^3^Н at the Shagan River were overlaid on sampling points and plotted on the map (Fig 3).

Results of the beta-spectrometric analysis showed that ^3^Н concentration in waters of the Shagan River varies widely from a value below the minimum detectable activity to 320,000 Bq/kg. The peak of ^3^H activity was recorded on a section of the river located at 5 km, and the minimum was recorded on a section at 70 km. At the confluence of the Shagan River waters and the Irtysh River, numerical values of ^3^Н are noted at the level of 90 Bq/kg (S2 Table).

**Fig 4:**
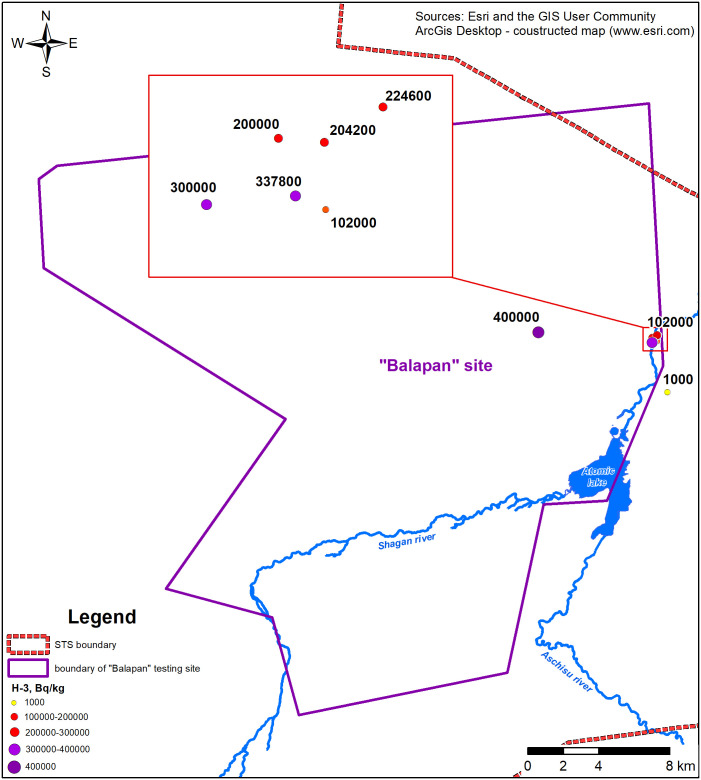
Beta-spectrometric measurements in groundwaters.

It should be noted that as a result of the nuclear tests conducted at this site, the underground environment is subject to the main radionuclide contamination. For example, concentrations of ^3^Н in groundwaters vary from 1,000 Bq to 400,000 Bq/kg (Fig 4) (S3 Table).

### 3.2. Discussion

**Fig 5:**
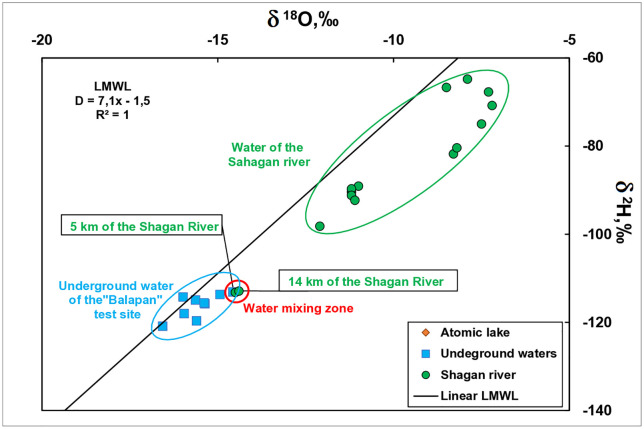
Correlation isotopic analysis.

To define surface and groundwater exchange, results of the stable δ^18^O and δ^2^H analysis were used. A correlation analysis was carried out (Fig 5).

According to the analysis carried out, groundwaters were revealed to contain ‘lighter’ isotopes compared to waters of the Shagan River. The obtained values for surface waters are located to the right of the meteoric water line, which may indicate the presence of fractionation processes, with the exception of the sections at 5 and 14 km.

The waters of sections 5 and 14 km are in the mixing zone with groundwater. This is indicated by identical isotope values of surface and groundwater. Thus, local spots were revealed in the riverbed with a groundwater outlet.

**Fig 6:**
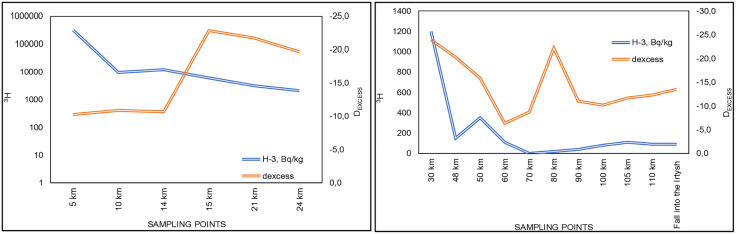
Distribution of ^3^Н and d_excess_ in Shagan River waters.

To understand the tritium contamination across the length of the Shagan River, ^3^Н and d_excess_ distribution was analyzed (Fig 6).

According to results of the correlation analysis, in sections 5, 10 and 14-km of the river d_excess_ varies from −10.3 to −10.9. This indicates the absence of evaporation processes in these waters. Such variation is directly associated with the groundwater entry. Groundwaters are not subject to fractionation processes, and d_excess_ varies from −3.9 to −9.7 (Table 1). In these sections, ^3^Н reaches maximum concentrations, namely, at 5 km its activity is equal to 320,000 Bq/kg. The assumption the inflow of groundwaters is consistent with earlier studies, which showed that these river sections have tectonic disturbances (faults) confined to the Zhanan shear zone, through which fracture-vein waters enter surface waters [34].

^3^Н concentrations in sections from 15 to 50-km range from 6,000 Bq/kg (15 km) to 350 Bq/kg (50 km). Values of d_excess_ vary from −22.9 (15 km) to −15.9 (50 km), which indicates some existing water evaporation processes and no additional recharge sources. The presence of man-made ^3^Н in this section is related to its transport by surface waters.

In sections 60 and 70-km, the d_excess_ value is noted to increase, which points to no evaporation processes. ^3^Н values are equal to 110 Bq/kg (60 km) and below the minimum detectable activity (70 km).

The section at 80-km, the d_excess_ value is noted to decrease, which indicates some existing evaporation processes (d_excess_ values reach −22.2). The minimum values of ^3^Н were recorded exactly in this section (20 Bq/kg) (Fig 6).

In other sections of the river, ^3^Н distribution is directly related to the evaporation coefficient. That is, if d_excess_ is greater than or equal to −10, then ^3^Н is noted to increase both 5, 10 and 14 km away from the Shagan River.

From the point of view of theoretical assessment, all sections of the river should be subject to the evaporation process equally, but this is not observed on the Shagan River. This indicates the entry of groundwater into surface water, as mentioned above, at 5 km.

**Fig 7:**
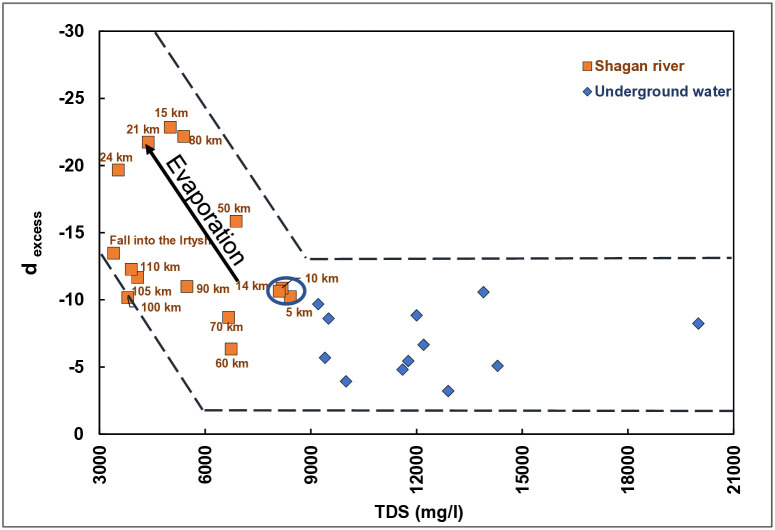
Interrelation between d_excess_ and salinity.

To confirm the abovementioned conclusion and determine additional sections of surface and groundwater mixing, the relationship between d_excess_ and the total salinity was analyzed (Fig 7).

Waters at 15, 21, 24, 80 and 110 km are subject to the greatest evaporation process, so d_excess_ is less than −10 and reaches a minimum value of −22.9. Water salinity at these points is greatly different from that of groundwaters. This is due to a missing water exchange at these points.

Water salinity in the 5, 10 and 14-km sections reaches 8,400 mg/l, which is close to values of groundwaters, ranging from 9,400 mg/l to 20,000 mg/l. Findings prove the interrelation between these sections and groundwaters. A mean value of d_excess_ is equal to −10.6, and this points to no evaporation processes in the period of interest.

Waters in 60 and 70-km sections of the Shagan River, as shown by outputs, are additionally recharged as d_excess_ is equal to −6.4 and −8.7, respectively. By salinity, these waters are dissimilar to groundwaters. For example, 60 and 70 km away, salinity varies from 5,390–6,665 mg/l. This may be due to the ‘hyporheic zone’ (underflow waters). The hyporheic zone is a porous space beneath and along the flow bed in which shallow groundwaters mix with surface waters. In summary, a portion of water from sections of a direct groundwater outlet (5, 10 and 14 km) is transported by surface waters and the rest is transported by underflow waters. In the 60 and 70 km sections, water is discharged from hyporheic zones to the surface in the form of springs. A similar picture can be noted at 110 km because the waters there are less exposed to evaporation compared to 80 km.

In summary, radioactive contamination of surface waters of the Shagan River is directly related to groundwater, due to its entry into the river bed.

## Conclusion

The study allowed us to identify key patterns of migration and spatial distribution of tritium-contaminated waters in the Shagan River bed, located within the former Semipalatinsk test site. The use of a set of isotopic (δ²H, δ¹^8^O, d_excess_) and hydrochemical (salinity) indicators in combination with measurements of tritium activity allowed us to deeply analyze the mechanisms of interaction between surface and groundwater and determine the main sources of radionuclide entry into the river system.

The results of the study showed that sections of the river located 5, 10 and 14 km from the river source show an intensive inflow of groundwater contaminated with tritium. These sections are characterized by elevated concentrations of the radionuclide, stable isotope values similar to groundwater, and increased mineralization, indicating direct interaction with deep aquifers.

In the river section from 15 to 50 km, water pollution with ^3^H is associated with the transfer of surface water by the river. Moderate levels of tritium and significant negative values of the d_excess_ are noted here, which indicates the prevalence of evaporation processes and the absence of additional recharge from underground sources. In the waters of the river sections of 60, 70 and 110 km, signs of water exchange with the hyporheic zone were recorded – an area located under the riverbed and along the banks of the river, where ground and surface waters mix. However, at 70 km of the river, the ^3^H concentration is below the minimum detectable activity, and at 60 and 110 km, the specific activity of tritium is 110 and 90 Bq/kg, respectively. At 80 km from the river source, the most distinct manifestation of evaporation processes is observed without signs of underground recharge. This is confirmed by minimal concentrations of tritium (20 Bq/kg) and significant negative values of the d_excess_ (about –22.2‰). At the confluence of the Shagan River and the Irtysh, the tritium activity is about 90 Bq/kg, which is significantly lower than the intervention level.

Thus, the conducted isotope-hydrochemical analysis allowed us to establish areas of intensive water exchange between underground and surface waters and to identify a hyporheic feeding zone that influences the formation of radionuclide contamination in the river system. The obtained data are an important scientific basis for further monitoring and development of environmental management strategies for water bodies exposed to radioactive contamination as a result of nuclear tests.

## Supporting information

S1 Fig Los Gatos Research LWIA 912−0008. (DOCX)

S2 Table Results of determination of the content of man-made radionuclide 3H in the waters of the Shagan River. (DOCX)

S3 Table Results of determination of the content of man-made radionuclide 3H in ground water ‘Balapan’ test site. (DOCX)
