# Supplementary figures and images for: Tritium contamination and hydrological transport in the Shagan River: An isotope hydrology study
Source: PLoS One. 2025 Oct 9;20(10):e0333260. doi: 10.1371/journal.pone.0333260 (PMC12510560; doi:10.1371/journal.pone.0333260)

| 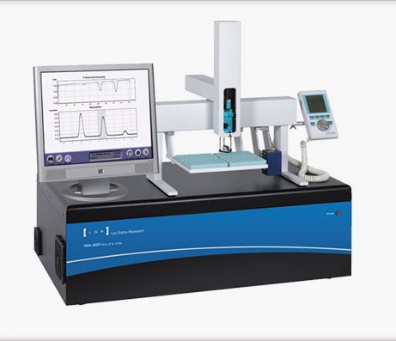 | 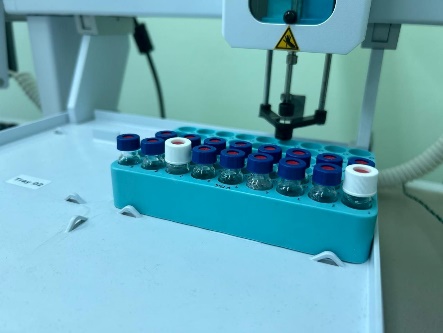 | 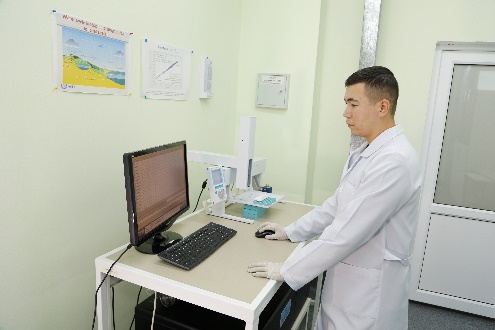 |
| --- | --- | --- |
| **S1 Fig. Los Gatos Research LWIA 912-0008** | | |

Supplement: S1 Fig — (DOCX) [file pone.0333260.s001.docx]
