# Supplementary material for: Tritium contamination and hydrological transport in the Shagan River: An isotope hydrology study
Source: PLoS One. 2025 Oct 9;20(10):e0333260. doi: 10.1371/journal.pone.0333260 (PMC12510560; doi:10.1371/journal.pone.0333260)
Supplement: S2 Table — (DOCX) [file pone.0333260.s002.docx]

**S2 Table. Results of determination of the content of man-made radionuclide ^3^H in the waters of the Shagan River**

| **Location of sampling** | **Sampling points** | **Month of sampling** | **Specific activity ^3^H, Bq/kg** |
| --- | --- | --- | --- |
| **Shagan river** | 5 km | september | 320 000 ± 32 000 |
| **Shagan river** | 10 km | august | 9 500 ± 950 |
| **Shagan river** | 14 km | september | 12 000 ± 1 200 |
| **Shagan river** | 15 km | august | 6 000 ± 600 |
| **Shagan river** | 21 km | august | 3 150 ± 315 |
| **Shagan river** | 24 km | august | 2 100 ± 210 |
| **Shagan river** | 50 km | august | 350 ± 35 |
| **Shagan river** | 60 km | august | 110 ± 11 |
| **Shagan river** | 70 km | august | < 6 |
| **Shagan river** | 80 km | august | 20 ± 6 |
| **Shagan river** | 90 km | august | 40 ± 6 |
| **Shagan river** | 100 km | august | 80 ± 8 |
| **Shagan river** | 105 km | august | 110 ± 11 |
| **Shagan river** | 110 km | august | 90 ± 9 |
| **Shagan river** | Fall into the Irtysh River | august | 90 ± 9 |
