# Supplementary material for: Tritium contamination and hydrological transport in the Shagan River: An isotope hydrology study
Source: PLoS One. 2025 Oct 9;20(10):e0333260. doi: 10.1371/journal.pone.0333260 (PMC12510560; doi:10.1371/journal.pone.0333260)
Supplement: S3 Table — (DOCX) [file pone.0333260.s003.docx]

**S3 Table. Results of determination of the content of man-made radionuclide ^3^H in ground water ‘Balapan’ test site**

| **Place of Sampling** | **Sampling points** | **Month of sampling** | **Specific activity ^3^H, Bq/kg** |
| --- | --- | --- | --- |
| **‘Balapan’ test site** | 53К | august | 1 000 ± 100 |
| **‘Balapan’ test site** | 5.5/4 | august | 200 000 ± 20 000 |
| **‘Balapan’ test site** | 4/4 | august | 300 000 ± 30 000 |
| **‘Balapan’ test site** | 1202-CS2 | august | 400 000 ± 40 000 |
| **‘Balapan’ test site** | 5 PS | august | 337 800 ± 33 780 |
| **‘Balapan’ test site** | 4/5 PS | august | 204 200 ± 20 420 |
| **‘Balapan’ test site** | 4/4 PS | august | 224 600 ± 22 460 |
| **‘Balapan’ test site** | 6 PS | august | 102 000 ± 10 200 |
